# An Improved Method of Maintaining Primary Murine Cardiac Fibroblasts in Two-Dimensional Cell Culture

**DOI:** 10.1038/s41598-019-49285-9

**Published:** 2019-09-09

**Authors:** Natalie M. Landry, Sunil G. Rattan, Ian M. C. Dixon

**Affiliations:** 1 Institute of Cardiovascular Sciences, St. Boniface Hospital Albrechtsen Research Centre, Winnipeg, Manitoba Canada; 2 Department of Physiology and Pathophysiology, Rady Faculty of Health Sciences, Winnipeg, Manitoba Canada; 3 Max Rady College of Medicine, University of Manitoba, Winnipeg, Manitoba Canada

**Keywords:** Biological techniques, Cell biology, Cell growth

## Abstract

Primary cardiac fibroblasts are notoriously difficult to maintain for extended periods of time in cell culture, due to the plasticity of their phenotype and sensitivity to mechanical input. In order to study cardiac fibroblast activation *in vitro*, we have developed cell culture conditions which promote the quiescent fibroblast phenotype in primary cells. Using elastic silicone substrata, both rat and mouse primary cardiac fibroblasts could be maintained in a quiescent state for more than 3 days after isolation and these cells showed low expression of myofibroblast markers, including fibronectin extracellular domain A, non-muscle myosin IIB, platelet-derived growth factor receptor-alpha and alpha-smooth muscle actin. Gene expression was also more fibroblast-like *vs*. that of myofibroblasts, as *Tcf21* was significantly upregulated, while *Fn1-EDA*, *Col1A1* and *Col1A2* were markedly downregulated. Cell culture conditions (eg. serum, nutrient concentration) are critical for the control of temporal fibroblast proliferation. We propose that eliminating mechanical stimulus and limiting the nutrient content of cell culture media can extend the quiescent nature of primary cardiac fibroblasts for physiological analyses *in vitro*.

## Introduction

The term “fibroblast” is assigned to a heterogeneous group of highly-motile stromal cells found in the interstitium, whose function and phenotype is tissue-dependent^1–3^. In the heart, cardiac fibroblasts serve to maintain tissue homeostasis and regulate extracellular matrix turnover. While there are no molecular markers that are entirely specific to cardiac fibroblasts, they are typically positive for transcription factor 21 (TCF21)^4–6^, vimentin^7–9^, and CD90 (or Thy-1)^10–12^. When subject to stress or injury, fibroblasts lose their normal phenotype and activate into myofibroblasts. Unlike quiescent fibroblasts, myofibroblasts are highly contractile and are characterized by a radically organized cytoskeleton featuring alpha-smooth muscle actin (αSMA) -positive stress fibers^13–16^, mature focal adhesions^17–19^, and increased production of periostin^20,21^ and fibrillar collagens^22,23^. In addition, the activation of cardiac myofibroblasts is also associated with alternative splicing of fibronectin, specifically denoted by the inclusion of the cell-associated extracellular domain A (ED-A)^24,25^.

Myofibroblast activation is a hallmark of cardiovascular disease, as these cells are responsible for the excessive deposition of extracellular matrix (ECM) proteins and are the primary drivers of fibrosis and its related pathologies^26,27^. Induction of the myofibroblast phenotype has been associated with a multitude of stimuli and the most common effector in this process is transforming growth factor-beta (TGF-β)^28,29^, which is associated with the initial inflammatory response after vascular or myocardial injury. Moreover, cardiac myofibroblasts are further driven to promote fibrogenesis in response to the autocrine and paracrine effects of other pro-inflammatory cytokines, such as platelet-derived growth factor (PDGF)^30^. Along with this response, cardiac myofibroblasts also exhibit a pronounced increase in PDGF receptor-alpha (PDGFRα) expression in disease states^31–33^. Hypertrophic agents such as growth factors and Angiotensin-II^34,35^, hyperglycemia^36,37^, and the presence of reactive oxygen species (ROS)^38,39^ have also been shown to contribute to this transition in phenotype. Finally, biomechanical input has also been implicated in myofibroblast activation. Isometric tension promotes the release of latent TGF-β present in the ECM^40,41^, and the formation of stress fibers with the incorporation of αSMA^42^ and this could be likened *in vitro* to seeding cells on stiff plastic surfaces. Similarly, mechanical loading and stretching modulates fibroblast function and phenotype, promoting the deposition of ED-A fibronectin^25^ and enhancing TGF-β signaling^43^. While it has traditionally been viewed as a permanent event, the activation of myofibroblasts has recently been observed as a reversible process in resident fibroblasts *in vivo*^5,44^.

Despite these findings, the mechanisms which govern the cardiac fibroblast and myofibroblast phenotypes are largely uncharacterized, as common cell culture techniques are not commensurate to physiologically-relevant conditions, and *in vivo* transgenic models are difficult to generate without affecting other stromal cells. Even when isolated from healthy myocardium, the spontaneous phenotype of primary cardiac fibroblasts in conventional cell culture is of pro-fibrotic, activated myofibroblasts within hours of plating^45^. However, it has been shown that culturing primary fibroblasts on elastic surfaces which are biomimetic to their native tissues can help to alleviate myofibroblast activation^46^. In the case of myocardium, culture surfaces with a compressibility, or elastic modulus (*E*), of ~7 kPa are representative of healthy tissue, while surfaces with *E* > 10 kPa are considered fibrotic^47,48^. This presents a considerable hurdle in that conventional polystyrene tissue culture plates are significantly stiffer, often upwards of *E* = 3 GPa^49,50^. In addition, while it is common practice to passage primary cells to promote homogeneity in the culture population, passaging further drives myofibroblast activation and prevents physiologically- pertinent studies^45^. This phenotypic plasticity presents a unique problem in that fibroblast physiology that is representative of healthy myocardium cannot be readily observed and manipulated *in vitro*.

In spite of the unstable nature of the cardiac fibroblast phenotype, the capacity to maintain these cells in a quiescent state in two-dimensional cell culture would enable much more accurate and reproducible means by which to study their physiology, and their response to genetic manipulation and pharmacological treatment. In this study, we present conditions in which unpassaged (P0) primary cardiac fibroblasts can be maintained for more than 72 hours *in vitro* without significant activation of the myofibroblast phenotype. These data support an alternative means by which to investigate the molecular and cellular physiology of primary cardiac fibroblasts *in vivo* studies.

## Results

### Myofibroblast markers are downregulated in low nutrient conditions with restricted biomechanical input

**Figure 1:**
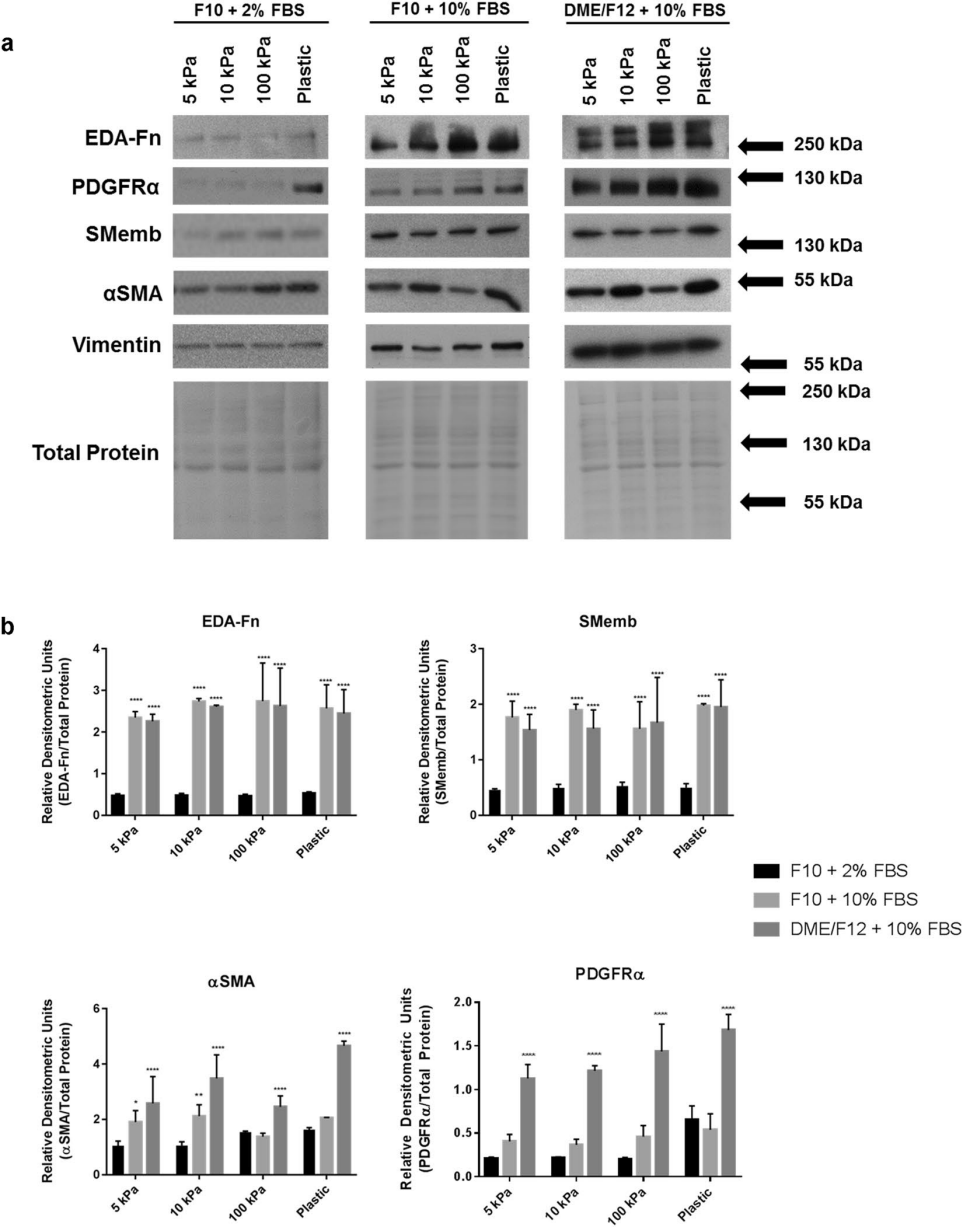
Myofibroblast marker expression in rat primary cardiac fibroblasts after >72 hours in culture. (**a**) Rat cardiac fibroblasts were plated on elastic plates with characteristic elastic moduli of 5 kPa, 10 kPa, and 100 kPa, with the indicated culture medium. Cells plated on conventional polystyrene tissue culture plates (rigid substrate – ranging from 30 to 100 MPa) were used as a comparative control. Protein from unpassaged primary cardiac fibroblasts was harvested ~3 days after plating. Vimentin expression was used as a pan-phenotypic control, and protein expression was normalized to total protein loading. (**b**) Graphical representation of data in (A). Data shown as the mean ± SD and is representative of n = 3–6 biological replicates. *P < 0.05, **P < 0.01, ***P < 0.005, ****P < 0.001 when compared to cells cultured in F10 medium with 2% FBS on the same substrate.

Mechanobiological properties (stiffness) of the ECM govern myofibroblast activation and function, and do so in the absence of input from TGFβ1/Smad signalling^50^. Moreover, myofibroblasts are contractile and sense and modulate stiffness within the ECM through focal adhesions via integrin binding^23^. In order to determine the physiological effects of two-dimensional cell culture on primary cardiac fibroblasts, we not only examined the influence of cell culture medium and serum, but also whether the compressibility of the culture surface was a greater factor in the spontaneous phenotype of the cells *in vitro*. After three days in culture, variable expression of myofibroblast markers ED-A fibronectin, non-muscle myosin heavy chain (SMemb or myosin IIB), and αSMA was observed in conditions of either low nutrient media (F10 with 2% FBS) or high nutrient media (DMEM/F12 with 10% FBS), plated on substrates that mimic the compressibility of healthy myocardium (5 kPa), or fibrosis-stiff substrate (100 kPa) (Fig. 1). On stiff substrate in combination with high serum, the preponderance of expression of myofibroblast markers is evident and significantly greater the other conditions tested. This increase was markedly evident with the expression of PDGRFα, which had a strong response to both substrate stiffness and medium composition. In mouse primary cardiac fibroblasts, we observed a significant increase in EDA-Fn, and PDGFRα, which was exhibited in concert with an increase in substrate stiffness (Supplemental Fig. 2). While conditions which favored the fibroblast phenotype were of F10 medium with low serum, it was evident that lower plate compressibility also imparted greater control of cell phenotype. The data supports the hypothesis that tuning the fibroblast substrate for reduced biomechanical input (ie. cells plated on elastic substrate versus stiff plastic) in combination with low nutrient conditions reduces the activation of fibroblasts cultured for extended periods.

### αSMA is excluded from the cytoskeleton in quiescent cardiac fibroblasts

**Figure 2:**
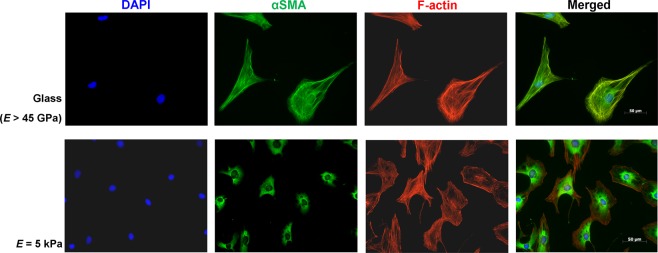
αSMA is excluded from F-actin stress fibers when cultured on 5 kPa culture surfaces. P0 rat cardiac fibroblasts were seeded at low confluency (<10%) on either glass (rigid substrate) or elastic coverslips and probed for αSMA (green) and F-actin (red) 3 days after plating. Images are representative of n = 3 independent biological replicates. Scale bar = 50 μm.

As αSMA is often viewed as the gold-standard marker for tissue fibrosis, and was evidently present in all samples we studied, we sought to compare its subcellular localization in our proposed *in* vitro model. Unpassaged primary rat cardiac fibroblasts were maintained *in vitro* for >72 hours; Fig. 2 provides comparative fields of cardiac fibroblasts plated on glass (rigid substrate) and fibroblasts plated on “cardiac soft” 5 kPa plates. F-actin and αSMA are double-stained in these fields, and the relative size of the cells in each set is remarkably different. αSMA is not incorporated into the cytoskeleton of the inactivated fibroblasts plated on the elastic substrata, indicating that these cells have not formed stress fibers, and are not actively contractile^51^. The merged fields of cells plated on glass show complete incorporation of αSMA into stress fibers, which reflects their phenotype of activated myofibroblasts.

### Cardiac fibroblast gene expression is further affected by biomechanical input, *in vitro*

**Figure 3:**
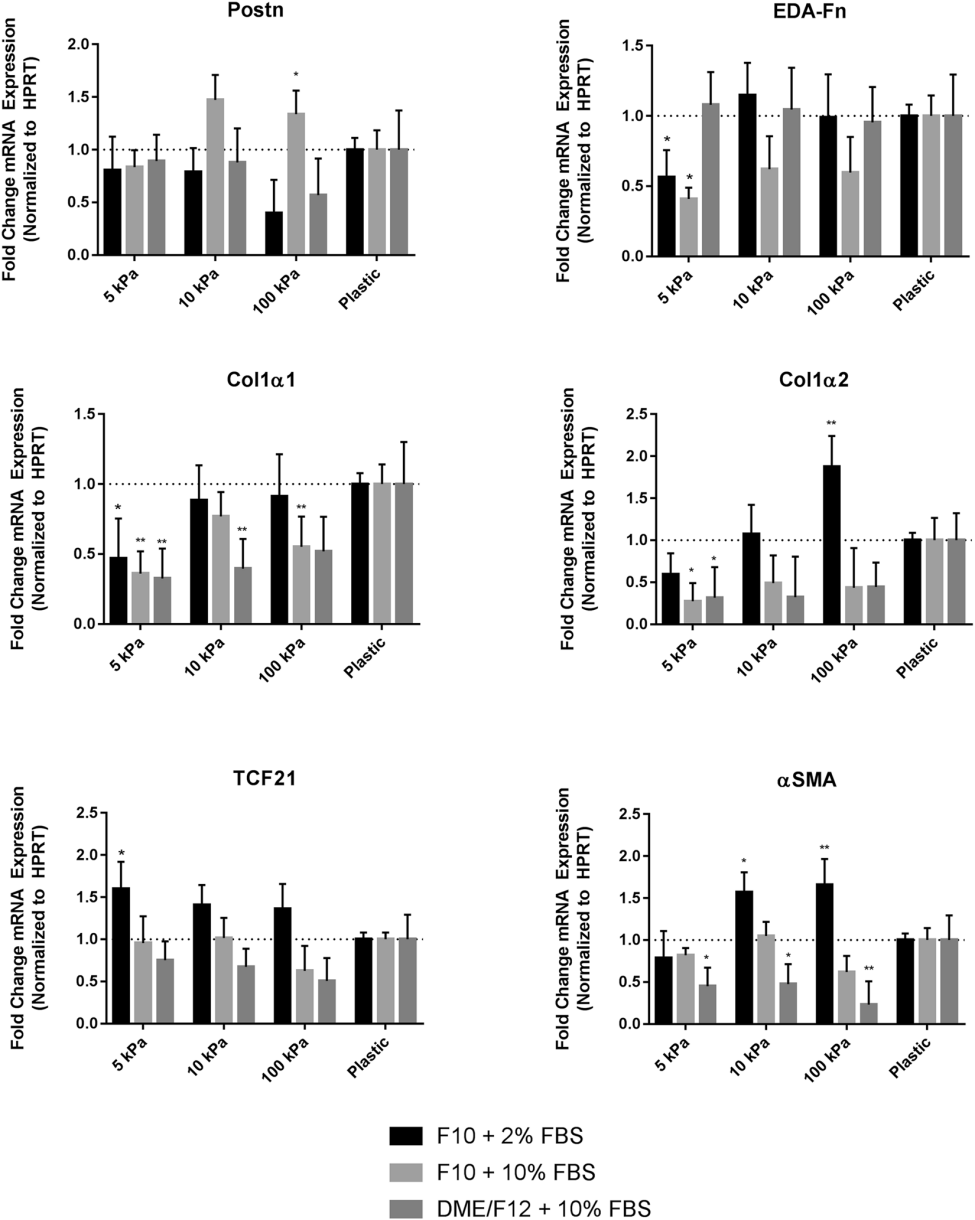
Fibroblast and myofibroblast gene expression in primary cardiac fibroblasts after >72 hours in culture. RNA was harvested from P0 rat cardiac fibroblasts 3 days after plating and used for qPCR. Samples from cells cultured on conventional plastic tissue (rigid substrate) culture surfaces were used as comparative controls. All reactions were performed in technical triplicates and were normalized to HPRT. Data shown as the mean ± SD and is representative of n = 3 biological replicates. ^*^P < 0.05, ^**^P < 0.01, when compared to cells cultured on plastic in the same culture medium.

In addition to protein expression, we sought to determine the effects of culture medium and/or substrate stiffness on gene expression in cultured primary cardiac fibroblasts. After three days *in vitro*, relative mRNA abundance of fibroblast- and myofibroblast-expressed genes including collagen type I monomers, αSMA, ED-A fibronectin, periostin (a secreted, matricellular protein), and Tcf21 were examined. Tcf21 is required for fibroblast formation, and is a nucleus-localized protein expressed in adult fibroblasts^52^, whereas periostin is a marker for activated fibroblasts^53,54^. We found *Tcf21* expression to be significantly elevated in the fibroblasts harvested from 5kPa substrate with F10 medium and 2% FBS, versus expression on plastic plates, whereas *Postn* was elevated in the 100 kPa plates relative to its expression on plastic plates in similar conditions (Fig. 3). ED-A fibronectin (*Fn1-EDA*) gene expression was significantly lower on 5 kPa plates versus the non-elastic plastic controls, whereas αSMA was elevated in 10 and 100 kPa plates versus plastic in low nutrient and low serum conditions. Collagen monomer (*Col1a1* and *Col1a2)* expression also shows similar responsiveness to plate stiffness, with expression increasing as substrate stiffness increases. While our results show some heterogeneity in the variable expression of marker mRNAs, which appears to be gene-dependent, the myofibroblast markers are generally upregulated in response to plating of fibroblasts on stiff substrates. When considering the effects of the cell culture medium, the conditions which resulted in the most inhibition of myofibroblast gene expression were those in F10 medium with 2% serum, although this effect can apparently be overridden by the compressibility of the tissue culture plate.

### Cardiac fibroblast proliferation can be limited in extended cell culture

**Figure 4:**
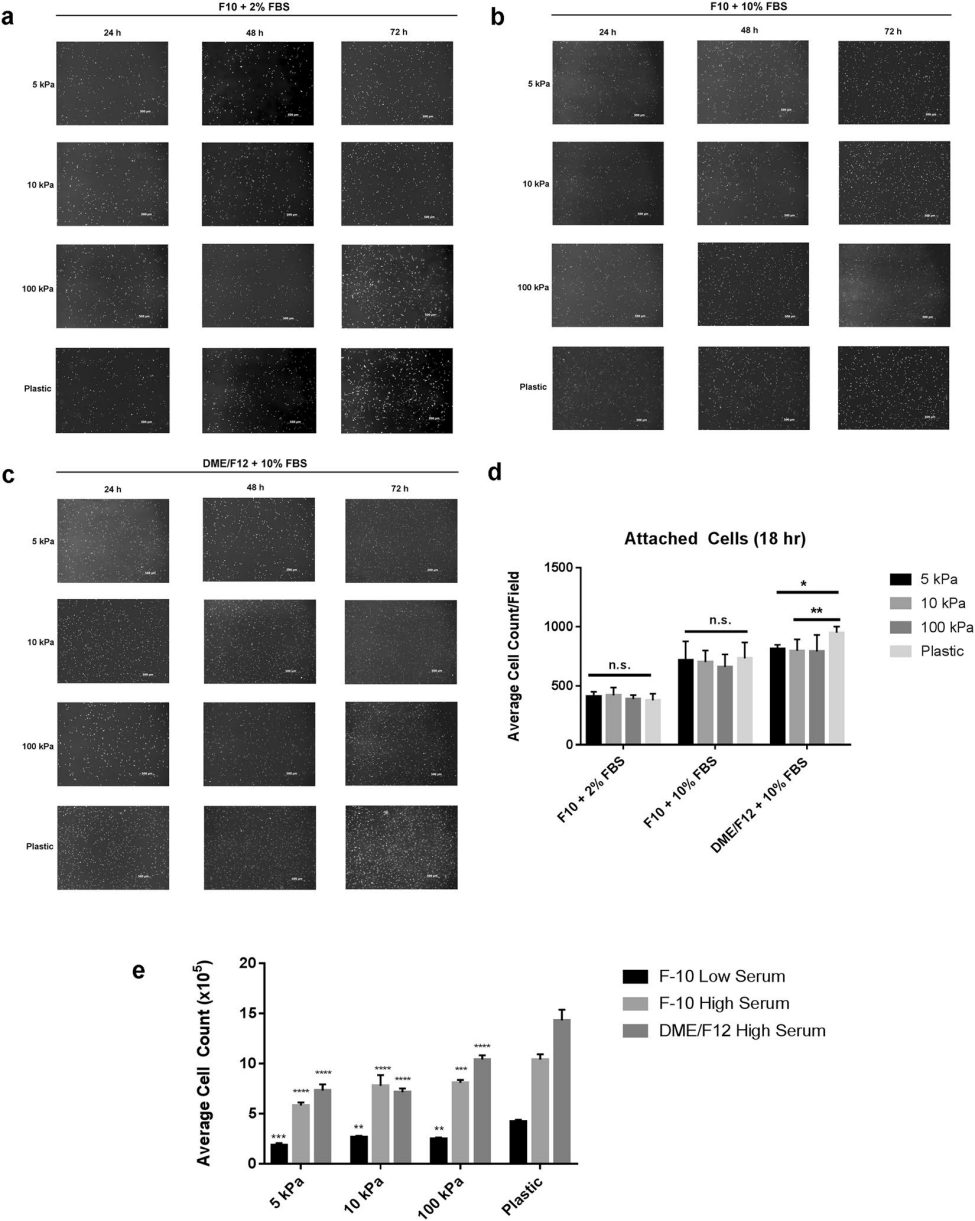
Nutrient restriction and decreased biomechanical input inhibits proliferation in primary cardiac fibroblasts. (**a**–**c)** P0 rat cardiac fibroblasts were plated on elastic plates with various elastic moduli, with the indicated culture medium, and stained with proliferation staining agent 24 hours after seeding. Images were captured immediately after staining and every 24 hours subsequently. Cells plated on conventional plastic tissue culture plates (rigid substrate) were used as a comparative control. Images are representative of n = 3 biological replicates. (**d**) Histrographical representation of cell counts 18 hours after plating and attachment (**e**) Histrographical representation of cell counts obtained after 3 days in culture (t > 72 hours). Data displayed as mean ± SD and is representative of n = 6 biological replicates. **P < 0.01, ***P < 0.005, ****P < 0.001 when compared to cells cultured on plastic in the same medium.

**Figure 5:**
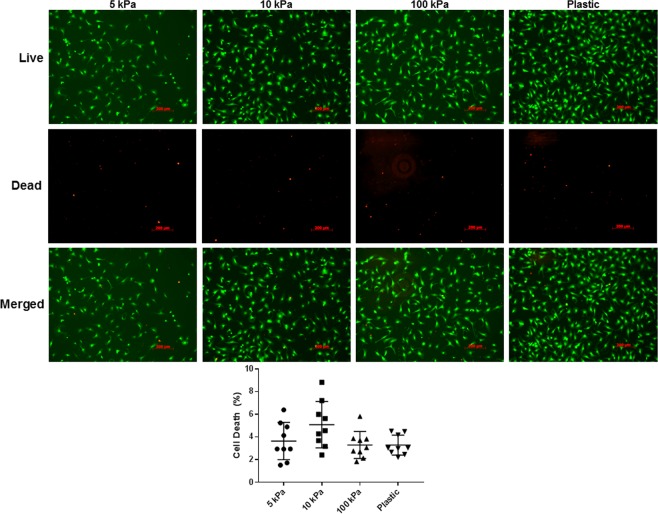
Assessment of cell viability in restricted nutrient conditions after >72 hours in culture. P0 rat cardiac fibroblasts were seeded onto substrata of various elastic moduli and cultured in F10 medium supplemented with 2% FBS for 4 days. Cell viability was assessed with Calcein-AM (green) staining, and cell death by ethidium homodimer (red). Each biological replicate was assessed by capturing 3 random fields at 10X magnification. Images are representative of n = 3 biological replicates, with 9 technical replicates each.

To further characterize isolated rat primary cardiac fibroblasts *in vitro*, we examined their proliferative behavior under various conditions. Specifically, we sought to investigate the responsiveness of fibroblast and myofibroblast proliferation in low and high serum conditions using media with low nutrient (F10) and high nutrient (DMEM/F12) content. In low serum conditions, cardiac fibroblast proliferation is suppressed by plating on 5 kPa, 10 kPa and 100 kPa plates with F10 medium, versus plastic controls (Fig. 4). A similar trend was observed using high serum conditions with the same medium, as well as with DMEM/F12; however the expansion of the cell population in conditions with DMEM/F12 was much more pronounced, especially on stiff plastic. To determine whether this observation was due to a difference in initial cell attachment between culture conditions, we counted the number of attached cells at 18 hours post-plating, after washing off any debris. We did not observe a significant difference in cell attachment when cells were cultured in low nutrient conditions however there was preferential attachment on stiffer culture surfaces when the cells were maintained in DMEM/F12 + 10% FBS. Additionally, to confirm that the observed lack of proliferation in F10 culture medium was not due to excessive cell death, at 96 hours post-plating the cells were stained with Calcein-AM (stains viable cells) and ethidium homodimer (stains nucleic acid/dead cells). We observed no significant difference in cell death between the various elastic moduli of the cell culture plates, suggesting that the compressibility of the culture substrata does indeed affect primary cardiac fibroblast activation and proliferation *in vitro* (Fig. 5). These results support the suggestion that proliferation of activated myofibroblasts is higher than inactive fibroblasts in culture, and that the state of activation can be controlled by both substrate stiffness and the nutrient content of the culture medium. Thus, we reveal an interaction between substrate stiffness, activation state of cardiac fibroblasts, and the proliferative capacity of primary cardiac fibroblasts *in vitro*.

## Discussion

Cardiac fibroblasts and activated myofibroblasts are component cells of the myocardium that contribute to the maintenance of the ECM in homeostatic hearts and to cardiac fibrosis after injury, respectively^3^. A novel interpretation of cardiac fibrosis (and other tissue fibrosis) is that rather than simply described as “over-active” wound healing, the organism may be attempting to utilize developmental programs that are typically active when generating functional muscle^23^. In the heart, fibroblasts are derived from different embryonic sources, with the majority from the epicardium (EMT contributing 80% of cells), and most of the remaining fibroblasts from the endocardium (EndoMT contributing 18%)^55^. Thus, defining these cells in a molecular context is difficult due to the lack of identification of a specific marker common to all fibroblasts. The mixed origin of cardiac fibroblasts notwithstanding, the common role of the activated myofibroblast is to generate and remodel the ECM^23^. Furthermore, the recent focus on cardiac fibrosis and the involvement of myofibroblasts and specific markers for them, including periostin, ED-A fibronectin, SMemb, and αSMA provides new impetus to direct fibrosis research to focus on activated myofibroblasts, which require refinements in approaches for their practical study^4,17,45^.

Accordingly, the estimation of the impact of fibroblast activation to myofibroblasts in the damaged and failing heart have become a much sought-after topic. Nonetheless, while the burgeoning number of scientific reports published during the past ten years reflects the overall acceptance of cardiac fibrosis as a player in the evolution of heart failure, methods to distinguish the activated from non-activated form of cardiac fibroblasts have not kept pace, with few exceptions. We, and other groups, have previously published studies including both unpassaged and passaged primary cardiac fibroblasts plated on stiff plastic substrata in an attempt to procure a baseline phenotype *in vitro*^56–58^. Despite these efforts, observing primary cardiac fibroblasts in conventional cell culture is limited to the first 12 to 16 hours before seeing overt activation of the myofibroblast phenotype^45^. As a result, the maintenance of the quiescent phenotype in culture has become a pressing issue, and is regularly overlooked in experimental design.

Herein we have provided detailed results to contextualize the importance of nutrients in culture media, culturing cells to eliminate biomechanical input, and minimize the impact of serum on fibroblast activation to allow for reliable culture of inactive cardiac fibroblasts. Upon examination of protein expression in rat primary cardiac fibroblasts maintained in culture for three days, it was evident that ED-A fibronectin, αSMA, and SMemb are differentially expressed among all conditions tested. The most differentially-expressed pro-fibrotic marker was PDGFRα, which had increased expression on stiff plastic substrata, and was even more highly-expressed in conditions of high nutrient and serum concentrations. A similar expression pattern for myofibroblast markers was observed in mouse cardiac fibroblasts, which were maintained in culture for a total of 10 days post-isolation (Supplemental Fig. 2). Recent evidence indicates that these markers may also serve to drive fibroblast activation, as myosin II may mediate myofibroblast activation in stiffened fibrotic lungs^59^, and that in the setting of a stiff matrix, αSMA incorporation into contractile stress fibers facilitates mesenchymal stromal cell fate by controlling YAP release^60^. In addition, PDGFRα has recently garnered interest as a marker of fibroblast activation and ECM remodeling, as its expression is upregulated in models of fibrosis an heart failure^30,31^. Along with the data presented here, the addition of PDGFRα to the gamut of markers often used to describe cardiac fibroblasts and myofibroblasts will provide a clearer understanding of a cell’s position on the spectrum spanning the two phenotypes. Very recently, the fibronectin ED-A domain has been shown to promote binding to latent TGF-β-binding protein-1 (LTBP-1), and thus enhances fibronectin-associated storage of TGF-β, which may then increase its availability for extracellular activation and stimulation of fibrosis^25^. The appearance of fibronectin ED-A domain remains a useful marker heralding the activation of fibroblasts to myofibroblasts, and their subsequent production and local accumulation of disordered ECM. In addition, we also observed more severe upregulation of all three markers, along with Hippo and EMT markers, when the elastic substrata were coated with soluble fibronectin (Supplementary Fig. 1). These findings corroborate current work which postulates that fibronectin is an ECM component drives fibrogenesis and heart failure^6^. Taken together, these results underscore the utility of ED-A fibronectin as a marker of phenotype plasticity in response to biomechanical and nutritive inputs in culture, and strengthen the case to use it as such in concert with αSMA incorporation into myofibroblasts’ stress fibers.

Beyond the expression of αSMA, its subcellular localization is paramount to its effects on cell phenotype. We observed not only that αSMA is indeed present in unpassaged fibroblasts on elastic substrates, but also that its incorporation into stress fibers is a greater indicator of the myofibroblast phenotype. Similar findings have been demonstrated in other stromal cell types, including subcutaneous^61^, dermal^59^, and hepatic portal fibroblasts^60^. Furthermore, αSMA can itself be a driving force in myofibroblast activation, as ectopic overexpression in stromal cells has been shown activate the myofibroblast phenotype, and generate a feed-forward loop in fibrogenesis^62^. It should also be noted that in the myocardium, αSMA is not only expressed by cells of mesenchymal origin, but can also be expressed by stressed or injured cardiomyocytes expressing fetal gene programs during active remodelling and dedifferentiation^53–55^. Collectively, these results suggest that αSMA cannot be entirely relied upon for accurate characterization of cardiac fibroblasts, and that its subcellular localization should also be considered when conducting *in vitro* cardiovascular studies.

**Figure 6:**
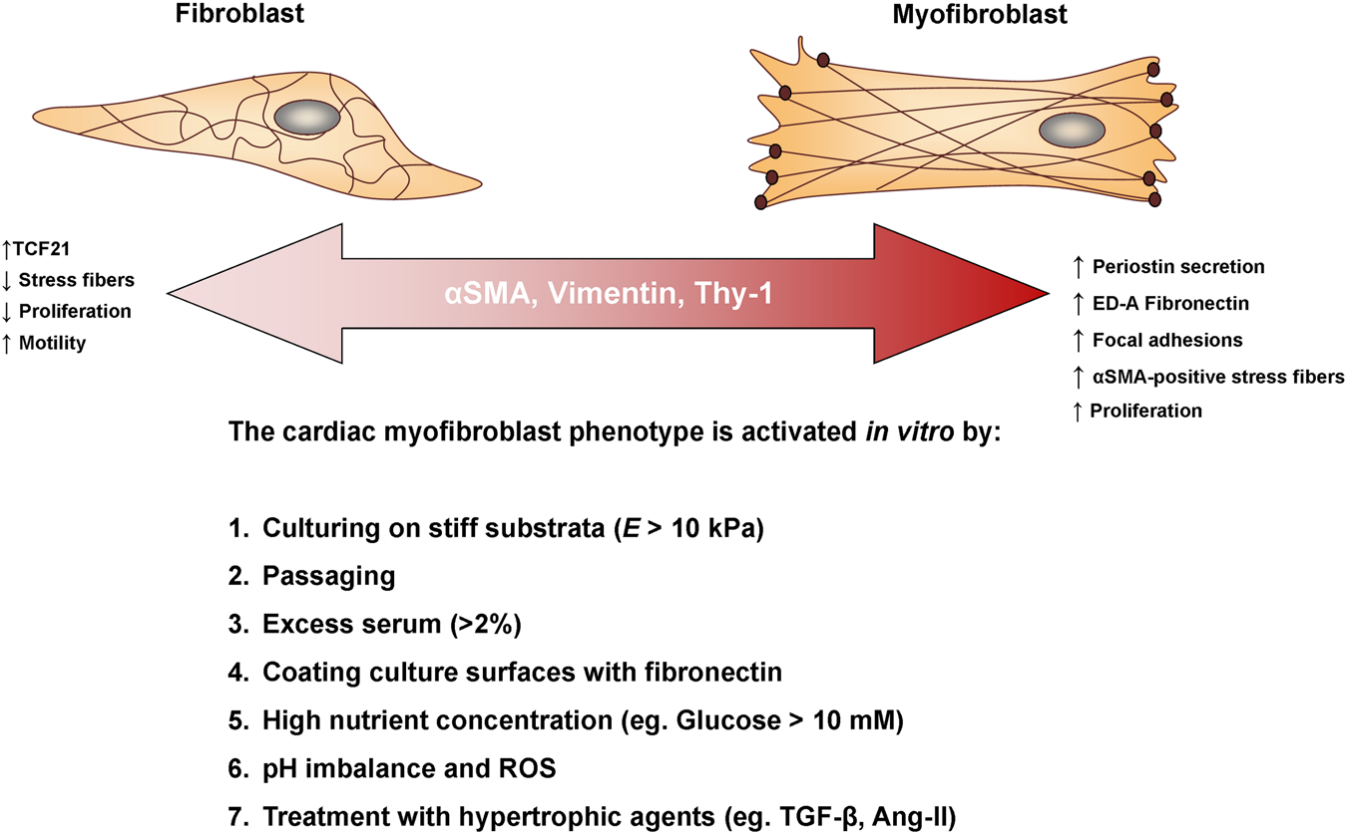
A schematic depicting the various factors affecting the fibroblast phenotype *in vitro*. Herein we summarize our main findings along with other known elements which influence primary cardiac fibroblast activation *in vitro*. We suggest that α-SMA is an ever-present marker on the cardiac fibroblast-myofibroblast spectrum, and that its incorporation in myofibroblast stress fibers is a key component of defining the activated phenotype. Previous studies on conventional plastic tissue culture plates have confirmed that passaging^45^, hyperglycemic-like conditions^67^, and treatment with ROS and hypertrophic agents indeed activate the myofibroblast phenotype and we conclude that excess serum also contributes to this activation.

Based on the findings of this study, we propose that relatively inactive primary cardiac fibroblasts can indeed be maintained *in vitro* for a period of time that is adequate for most molecular assays, provided that certain parameters (ie. passage number, culture medium, plate compressibility) are addressed (Fig. 6). The majority of published literature which implements primary cardiac cell culture, including those previously published by our lab, do not take into account the biomechanical, nutritional, and hormonal input to which the cells are subject in two-dimensional culture. Likewise, primary cardiac fibroblasts which are subject to passaging are also often used in order to maximize cell numbers and decrease the number of animals or tissue specimens for a given experiment. This is often seen as reasonable as it is simple, convenient, and offers some flexibility to the type of molecular assays employed in fibroblast-centric research. Using the methods described here, fewer cells are required to seed plates and passaging is not used; this can significantly decrease the material requirements for a given experiment. While diverging from traditional cell culture methods is not easily accomplished from a technical perspective, it is essential to consider all factors when designing experiments and interpreting data, especially as current *in vitro* studies are still using what would be considered as activated myofibroblasts. It is known that cardiac fibroblasts exist on a delicate phenotypic spectrum that is highly reactive to the extracellular environment and methods should be adapted to generate accurate and reproducible results. This will facilitate the generation of data which is relevant to physiological conditions, but will also promote rigor and consistency in the literature. The ability to control myofibroblast activation greatly improves the sensitivity of genetic and pharmacological assays. Future exploration into medium composition (eg. serum-free media) would certainly enable further refinements on this method. Thus, to better understand the pathogenesis of cardiac fibrosis and the effects of potential therapeutic interventions, the consequences of *in vitro* conditions on cell physiology should be carefully considered.

## Materials and Methods

### Animal ethics

All experimental protocols involving live animals were reviewed and approved by the University of Manitoba Animal Care Committee, and were generated in accordance with the standards of the Canadian Council of Animal Care.

### Preparation of elastic tissue culture surfaces

PrimeCoat silicone elastic tissue culture plates (10 cm) and coverslips (ExCellness Biotech SA, Lausanne, Switzerland) were coated with 10 μg/mL porcine gelatin type A (0.2 mL/cm^2^) in sterile water overnight at 37 °C, 5% CO_2_. Prior to plating cells, the supernatant was removed and replaced with complete culture medium. Polystyrene (non-elastic plastic) tissue culture dishes and glass coverslips were also treated in a similar manner and used as comparative controls when evaluating various elastic moduli.

### Isolation of rat primary cardiac fibroblasts

Rat primary cardiac fibroblasts were isolated, as previously described^56,57,63^ with some modifications. Male Sprague-Dawley rats weighing 101–125 g were anaesthetized with a ketamine-xylazine cocktail (100 mg/kg ketamine; 10 mg/kg xylazine) via intraperitoneal injection. Upon loss of limb reflexes, heparin (6 mg/kg) was administered intravenously via the femoral artery. Hearts were excised and briefly placed in Dulbecco’s Modified Eagle’s medium/Ham’s F12 nutrient mixture (DMEM/F12) prior to cannulating via the aorta on a Langendorff apparatus. The hearts were then subject to retrograde perfusion with DMEM/F12, followed by Minimum Essential Medium, Spinner’s Modification (S-MEM) to cease cardiac contraction and promote cell dissociation. Finally, the hearts were perfused with S-MEM supplemented with 640 U/mL collagenase type II (Worthington Biochemical Corporation, Lakewood, NJ) with recirculation for 25 minutes. Once digested, the tissue was incubated at 37 °C, 5% CO_2_ for 10 minutes before neutralizing the collagenase with 10 mL of DMEM/F12 supplemented with 2% fetal bovine serum (FBS) and further dissociated by trituration with a serological pipette. The resulting cell suspension was then passed through a 40 μm sterile cell strainer (Thermo Fisher Scientific, Waltham, MA) to remove any undigested tissue and debris. The cells were pelleted by centrifugation at 200 × *g* for 7 minutes, and re-suspended in 45 mL of complete cell culture medium. Three different types of media were used for comparison: F10 with 2% FBS, F10 with 10% FBS, and DMEM/F12 with 1 μM ascorbic acid and 10% FBS. All media was supplemented with 100 U/mL penicillin-streptomycin. For each 10 dish, 3 mL of cell suspension was added to a total of 10 mL of medium at plating. For coverslips in 35 mm or 6-well dishes, 0.5 mL of cell suspension was added to a total of 2 mL medium per dish or well. Fibroblasts were allowed to adhere for 2.5 hours at 37 °C, 5% CO_2_; cultures were then briefly washed twice with phosphate buffered saline (PBS; pH 7.4) supplemented with penicillin-streptomycin and then fresh complete culture medium was added. The following day, the cultures were once again washed twice with PBS, and the growth medium was replaced. The culture medium was subsequently replaced once per day until harvesting.

### Isolation of mouse primary cardiac fibroblasts

Primary mouse cardiac fibroblasts were isolated using a modified version of a previously-published protocol^64^. In short, 8- to 12-week old male C57BL/6 mice were anaesthetized with 3% isofluorane until loss of limb reflexes. The chest was opened to excise the heart with a portion of the ascending aorta remaining attached. The heart was promptly flushed with 10 mL of EDTA buffer (5 mM EDTA, 130 mM NaCl, 5 mM KCl, 500 nM NaH_2_PO_4_, 10 mM HEPES, 10 mM Glucose, 10 mM 2,3-Butanedione 2-monoxime, and 10 mM Taurine, pH 7.8), followed by 3 mL of perfusion buffer (1 mM MgCl_2_, 130 mM NaCl, 5 mM KCl, 500 nM NaH_2_PO_4_, 10 mM HEPES, 10 mM Glucose, 10 mM 2,3-Butanedione 2-monoxime, and 10 mM Taurine, pH 7.8) using a 27-gauge needle inserted into the ventricles via the apex. While still injecting through the apex, the heart was perfused twice with 25 mL S-MEM supplemented with collagenase type II (330 U/mL), while collecting the solution in a 10 cm tissue culture dish. Once the heart was sufficiently digested, it was gently pulled apart with forceps and allowed to digest in 20 mL of the collagenase solution at 37 °C, 5% CO_2_ for another 10 minutes. After triturating the tissue, the resulting suspension was neutralized with 10 mL of complete culture medium and passed through a 40 μm cell strainer and pelleted by centrifugation, as mentioned above. The resulting pellet was resuspended in 40 mL of DMEM/F12 supplemented with 10% FBS, 1 μM ascorbic acid, and 100 U/mL penicillin-streptomycin and the cells were then evenly distributed among 4–10 cm dishes and allowed to adhere for 3 hours. Adherent cells were then gently washed once with pre-warmed PBS (pH 7.4) supplemented with antibiotics, and the DMEM/F12 growth medium was replaced. The culture medium was replaced in a similar fashion every 24 hours until they adopted a spindle-shaped morphology (~3–4 days), after which the medium was switched to F10 supplemented with 2% FBS and Insulin-Transferrin-Selenium-Sodium Pyruvate (ITS-A; Thermo Fisher). The cells were harvested once they reached 40–50% confluency, at approximately 10 days post-isolation.

### Cell proliferation imaging and counting

Approximately 18 hours after plating, unpassaged (P0) fibroblast cultures were washed twice with PBS. Pre-warmed, serum-free and antibiotic-free medium was then supplemented with Cytopainter Green Cell Proliferation Agent (Abcam, Cambridge, UK). Cells were treated with the dye solution at 37 °C, 5% CO_2_ for 30 minutes, protected from light. The dye solution was removed, and the cells were briefly washed twice with PBS before replacing with complete culture medium. The cells were imaged every 24 hours post-plating with a Zeiss LSM 5 Pascal microscope using 4X and 10X objectives and an excitation wavelength of 488 nm. Images were processed using AxioVision Microscopy software (Zeiss, rel. 4.8).

Initial cell counts at 18 hours post-plating were performed by using phase contrast microscopy images and ImageJ software^65^, using three randomly-selected fields for each biological replicate, totaling 9 technical replicates for each cell culture condition. Manual cell counting was accomplished using a Moxi Z automated cell counter (Orflo Technologies, Ketchum, ID). Cells were trypsinized and re-suspended in an excess volume of complete culture medium, and a 1:10 dilution of the cell suspension was used for each count. Two counts were taken for each biological replicate to ensure accuracy for each measurement. Manual cell counting was performed in triplicate for each time point and cell proliferation imaging was accomplished by selecting three randomly-chosen fields for each cell culture condition.

### Protein isolation

After approximately 80 hours after plating, cells were trypsinized and pelleted by centrifugation at 200 × *g* for 5 minutes. Cell pellets were then washed with PBS and re-pelleted by centrifugation for 3 minutes. The supernatant was removed and the pellets were lysed with RIPA lysis buffer supplemented with protease inhibitor cocktail (P8340; Sigma-Aldrich Canada Co., Oakville, ON) and phosphatase inhibitors (10 mM NaF, 1 mM Na_3_VO_4_, and 10 mM EGTA). The resulting lysates were then vortexed and incubated on ice for 30 minutes. Following incubation, the lysates were briefly sonicated for 5 seconds, and then centrifuged at 16 000 × *g* for 15 minutes at 4 °C. Supernatants were transferred to new microcentrifuge tubes and protein concentrations were determined using a bicinchoninic acid (BCA) assay.

### Immunoblotting

SDS-PAGE of 25 μg of protein was performed on 8% reducing gels. Proteins were transferred at 4 °C onto PVDF membranes in tris-glycine buffer containing 20% methanol. Total protein loading was measured prior to blotting using Ponceau S staining and densitometric analysis. Non-specific binding sites were blocked with 5% skim milk in tris-buffered saline supplemented with 0.1% Tween-20 (TBS-T) at room temperature. The blots were thoroughly washed in TBS-T before applying primary antibodies overnight at 4 °C with shaking. Primary antibodies were used at the following dilutions: ED-A (cellular) fibronectin (1:1000; MAB1940; MilliporeSigma, Burlington, MA; or NBP1-91258; Novus), SMemb (1:1000; ab684; Abcam), αSMA (1:5000; A2547; Sigma). Because the primary fibroblasts originated from a heterogenous population of cells, vimentin (1:2000; ab8069; Abcam) and platelet-derived growth factor receptor alpha (PDGFRα; 1:1000; ab134123; Abcam) were used as a phenotype controls on each blot. Appropriate HRP-conjugated secondary antibodies (Jackson ImmunoResearch, West Grove, PA) were applied at a 1:5000 dilution for 1 hour at room temperature. Protein detection was done using ECL substrate, and protein bands were visualized on blue X-ray film. Protein expression was measured by relative densitometry using Quantity One® analysis software (version 4.6.9; Bio-Rad).

### RNA isolation and quantitative PCR

Primary cardiac fibroblasts were isolated by trypsinization and centrifugation at 200 × *g* for 5 minutes. Column-based RNA isolation was performed using the PureLink® RNA Mini kit (Invitrogen, Carlsbad, CA) according to the manufacturer’s instructions. Approximate RNA concentration and purity was assessed by measuring the absorbance at 260 and 280 nm using a NanoDrop™ Lite Spectrophotometer (Thermo Scientific).

Two-step qPCR was performed first by synthesizing cDNA from 100 ng of RNA, using the Maxima™ First Strand cDNA synthesis for RT-qPCR (Thermo Scientific), and included initial treatment with dsDNase. Amplification reactions were prepared according to the Luna® Universal qPCR Master Mix (New England Biolabs, Ipswich, MA) protocol, using 1 μL of of cDNA template and 200 nM of forward and reverse primers in a final volume of 10 μL. PCR amplification was performed in triplicate for each reaction on a QuantStudio 3 Real-Time PCR System (Applied Biosystems, Foster City, CA) using the fast cycling mode. The following cycling program was used: initial denaturation at 95 °C (60 seconds), followed by 40 cycles of denaturation at 95 °C (15 seconds) and extension at 60 °C (30 seconds). After amplification, a continuous melt curve was generated from 60 °C to 95 °C. Relative gene expression was calculated using the 2^−ΔΔCt^ method^66^, using the samples from cells plated on plastic as controls for each sample set, and normalized to HPRT. Primer pairs and their corresponding targets are illustrated in Table 1.

**Table 1:** List of primer pairs used in quantitative PCR.

Gene	Accession	Forward Primer (5′-3′)	Reverse Primer (5′-3′)
*Acta2*	NM_031004.2	AGATCGTCCGTGACATCAAGG	TCATTCCCGATGGTGATCAC
*Col1a1*	NM_053304.1	TGCTCCTCTTAGGGGCCA	CGTCTCACCATTAGGGACCCT
*Col1a2*	NM_053356.1	TGACCAGCCTCGCTCACAG	CAATCCAGTAGTAATCGCTCTTCCA
*Fn1*	NM_019143.2	ACTGCAGTGACCAACATTGACC	CACCCTGTACCTGGAAACTTGC
*Hprt1*	NM_012583.2	CTCATGGACTGATTATGGACAGGAC	GCAGGTCAGCAAAGAACTTATAGCC
*Postn*	NM_001108550.1	GCTTCAGAAGCCACTTTGTC	CGCCAACTACATCGACAAGG
*Tcf21*	NM_001032397.1	CATTCACCCAGTCAACCTGA	CCACTTCCTTTAGGTCACTCTC

### Fluorescence immunocytochemistry

Primary cardiac fibroblasts were seeded at a low confluency onto either glass coverslips in 6-well dishes, or elastic (*E* = 5 kPa) silicone coverslips (ExCellness) in 35 mm dishes, coated with porcine gelatin type A, and maintained in culture with F10 medium with 2% FBS for 72 hours. The cells were briefly washed in PBS and fixed in 4% paraformaldehyde for 15 minutes at room temperature. After another brief wash in PBS, the cells were permeabilized with 0.1% Triton X-100 in PBS for 15 minutes, and non-specific binding sites were blocked for 1 hour in 5% normal goat serum (Invitrogen) in PBS. The blocking agent was removed by another set of washes before applying primary antibodies diluted in 1% bovine serum albumin (BSA) in PBS. αSMA was probed using a 1:50 dilution (A2547; Sigma) and incubated overnight at 4 °C in a humidified chamber. The following day, the cells were thoroughly washed three times in PBS and incubated with Alexa Fluor 488-conjugated secondary antibody (1:500; A27023; Invitrogen) for 1 hour at room temperature. After a brief wash in PBS, F-actin was stained using a 1:500 dilution of rhodamine-phalloidin (R415; Invitrogen) in PBS. After several washes over a period of 30 minutes, the coverslips were thoroughly dried using gentle suction and mounted on glass slides using Fluoroshield™ mounting medium with DAPI (Abcam) and allowed to cure at room temperature for 24 hours. Cells were imaged using a Zeiss LSM 5 Pascal microscope as described above, using DAPI, FITC and Texas Red detection channels.

### Cell viability assay

The viability of cells in each culture condition was assessed after 96 hours in culture. Cells were washed twice with pre-warmed PBS, then treated with 2 μM Calcein-AM (C3100, Thermo Fisher) and 2.5 μM ethidium homodimer (E1169, Thermo Fisher) in PBS for 30 minutes at 37 °C, 5% CO_2_. The stains were then gently removed by aspiration and replaced with fresh PBS. Cells were imaged immediately, using FITC and Texas Red detection channels.

### Data analysis and statistics

Statistical analyses and graphs were generated using Graph Pad Prism 7. All data are presented as the mean ± standard deviation, unless otherwise indicated in figure legends. Individual biological replicates are counted as one experiment involving cells from only one animal. Grouped data analyses were performed using one-way or two-way ANOVA followed by Tukey’s post-hoc test, with significance recorded if *P* < 0.05.

## Supplementary information

Supplementary Files
