# Supplementary material for: An Improved Method of Maintaining Primary Murine Cardiac Fibroblasts in Two-Dimensional Cell Culture
Source: Sci Rep. 2019 Sep 9;9:12889. doi: 10.1038/s41598-019-49285-9 (PMC6733858; doi:10.1038/s41598-019-49285-9)

An Improved Method of Maintaining Primary Murine Cardiac Fibroblasts in Two-Dimensional Cell Culture

Natalie M. Landry, Sunil G. Rattan, and Ian M.C. Dixon*

Institute of Cardiovascular Sciences, St. Boniface Hospital Albrechtsen Research Centre,

Department of Physiology and Pathophysiology, Rady Faculty of Health Sciences,

Max Rady College of Medicine, University of Manitoba, Winnipeg, Canada

*Address for correspondence:

Ian M.C. Dixon, PhD, FIACS
Professor, Department of Physiology and Pathophysiology
St. Boniface Hospital Albrechtsen Research Centre
R3010 - 351 Taché Avenue,
Winnipeg, MB, R2H 2A6, Canada
Ph: 204-235-3419 Fax: 204-233-6723
Email: idixon@sbrc.ca
ORCID: https://orcid.org/0000-0002-3763-5961

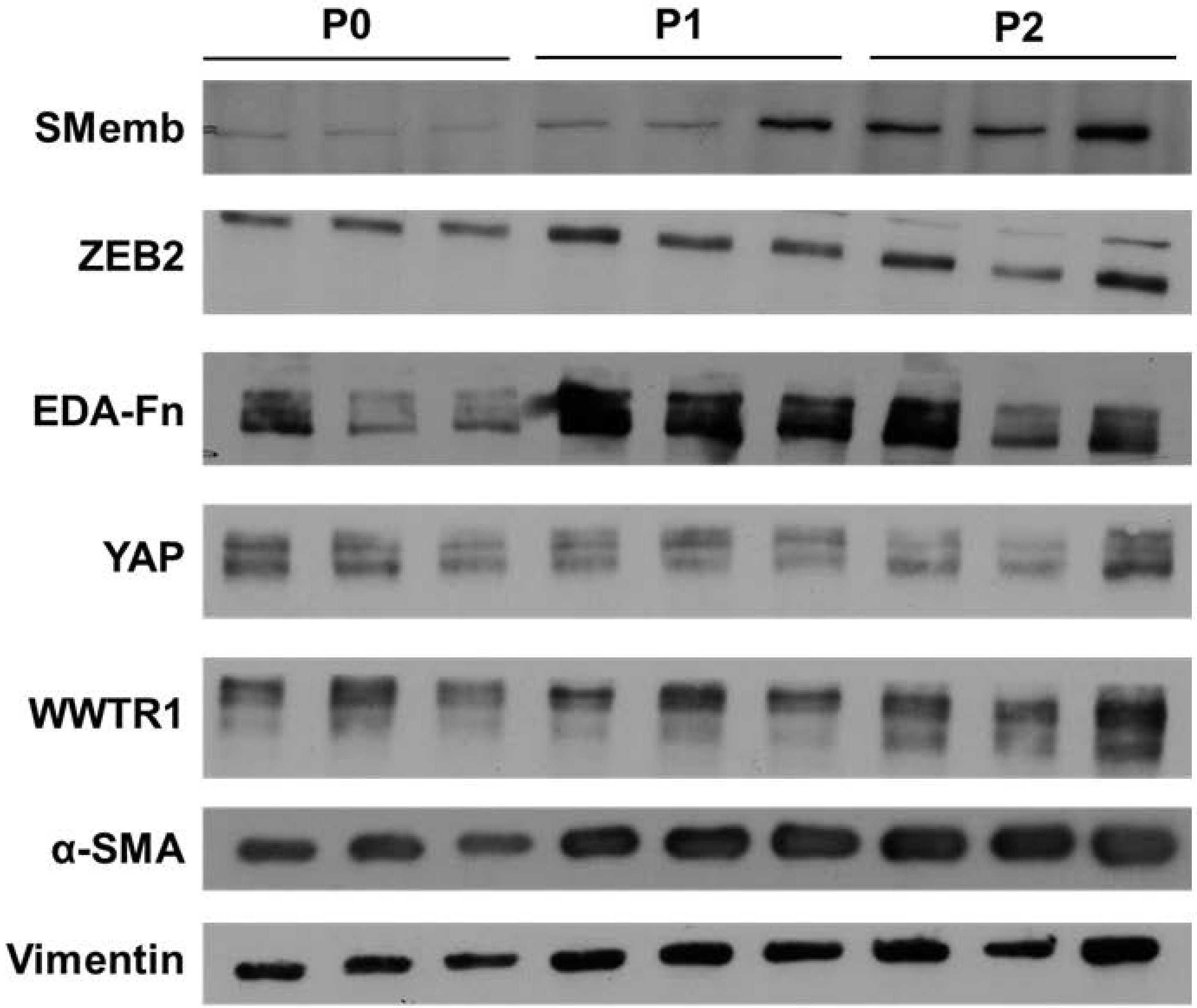
P0
P1
P2
SMemb
ZEB2
EDA-Fn
YAP
WWTR1
α-SMA
Vimentin

Supplementary Figure 1. Protein expression pattern of unpassaged and passaged primary cardiac fibroblasts on 5 kPa elastic plates coated with fibronectin. P0 rat cardiac fibroblasts were seeded onto elastic plates coated with 2 ug/cm2 human plasma fibronectin in F10 cell culture medium supplemented with 10% FBS. Cells were cultured for 48 hours prior to passaging (P1) onto new 5 kPa plates and this was repeated once to P2 and protein was harvested for immunoblotting. Vimentin was used as a phenotype control. Data shown is of n = 3 independent biological replicates. ZEB2, Zinc finger E-box Binding homeobox 2; YAP, Yes-Associated Protein; WWTR1, WW domain-containing Transcriptional co-Regulator 1.

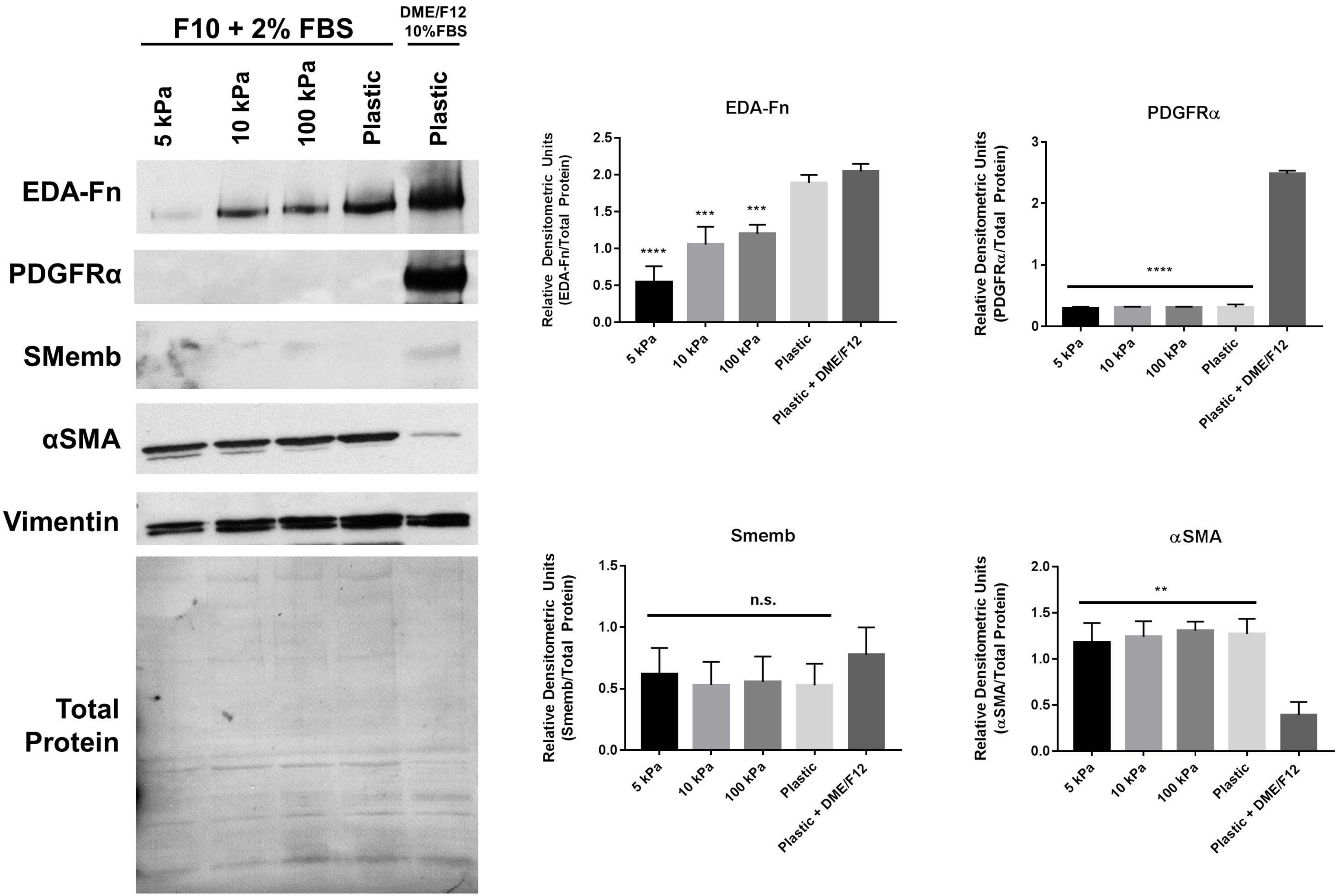

F10 + 2% FBS
DME/F12 10%FBS
5 kPa
10 kPa
100 kPa
Plastic
Plastic
EDA-Fn
PDGFRα
SMemb
αSMA
Vimentin
Total Protein
EDA-Fn
Relative Densitometric Units (EDA-Fn/Total Protein)
****
***
***
PDGFRα
Relative Densitometric Units (PDGFRα/Total Protein)
****
Smemb
Relative Densitometric Units (Smemb/Total Protein)
n.s.
αSMA
Relative Densitometric Units (αSMA/Total Protein)
**
5 kPa
10 kPa
100 kPa
Plastic
Plastic + DME/F12

Supplementary Figure 2. Myofibroblast marker expression in mouse primary cardiac fibroblasts after 10 days in culture. Mouse primary cardiac fibroblasts were isolated from 8-12 week old male C57BL/6 mice and plated on culture substrata of varying elastic moduli. One non-elastic plastic plate per replicate remained in DMEM/F12 + 10% FBS for the duration of the study as a comparative control, while the remaining plates were cultured in F10 + 2% FBS + ITS from 3 days post-isolation onward. Protein from unpassaged primary cardiac fibroblasts was harvested 10 days after plating, or once they reached 40-50% confluency. Vimentin expression was used as a pan-phenotypic control, and protein expression was normalized to total protein loading. n.s., not significant; $^{**}P < 0.01$, $^{***}P < 0.005$, $^{****}P < 0.001$, when compared to cells cultured on stiff plastic in DMEM/F12 + 10% FBS.

Table 1: List of primers used in quantitative PCR

Gene	Gene Product	Accession	Forward Primer (5'- 3')	Reverse Primer (5'-3')
Acta2	αSMA	NM_031004.2	AGATCGTCCGTGACATCAAGG	TCATTCCCGATGGTGATCAC
Col1a1	Collagen Type I Alpha 1 Chain	NM_053304.1	TGCTCCTCTTAGGGGCCA	CGTCTCACCATTAGGGACCCT
Col1a2	Collagen Type I Alpha 2 Chain	NM_053356.1	TGACCAGCCTCGCTCACAG	CAATCCAGTAGTAATCGCTCTTCCA
Fn1	Fibronectin (ED-A Splice Variant)	NM_019143.2	ACTGCAGTGACCAACATTGACC	CACCCTGTACCTGGAAACTTGC
Hprt1	Hypoxanthine-Guanine Phosphoribosyltransferase	NM_012583.2	CTCATGGACTGATTATGGACAGGAC	GCAGGTCAGCAAAGAACTTATAGCC
Postn	Periostin	NM_001108550.1	GCTTCAGAAGCCACTTTGTC	CGCCAACTACATCGACAAGG
Tcf21	TCF21	NM_001032397.1	CATTCACCCAGTCAACCTGA	CCACTTCCTTTAGGTCACTCTC

F10 + 2% FBS

EDA-Fn

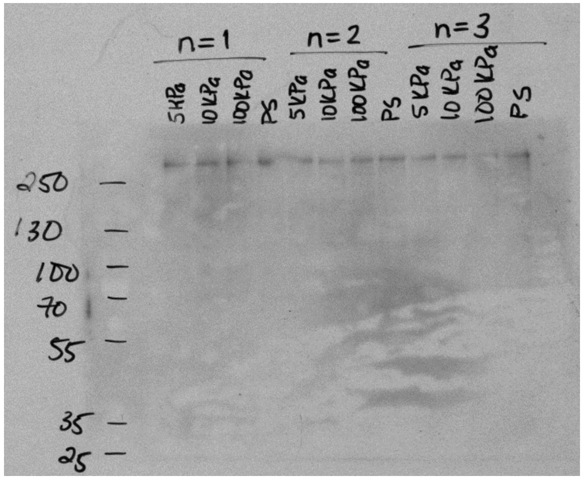

SMemb

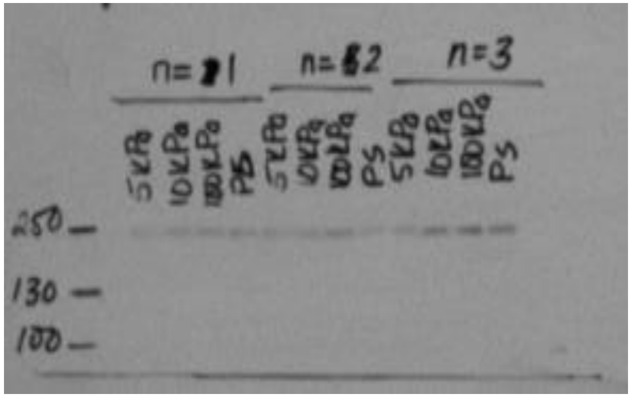

αSMA

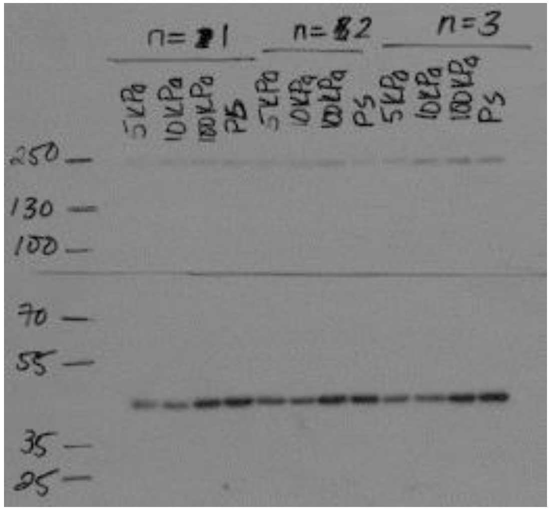

Vimentin

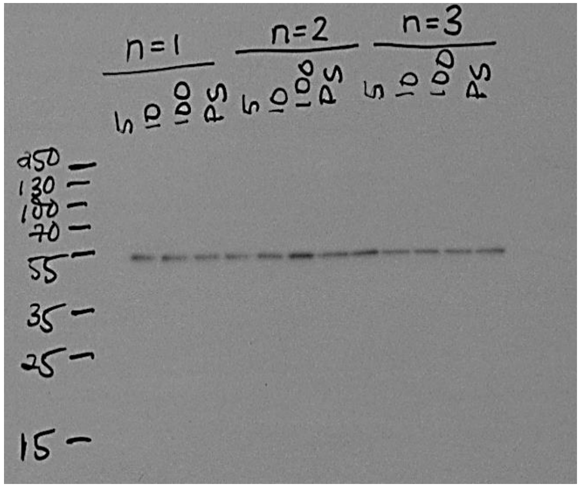

PDGFRα

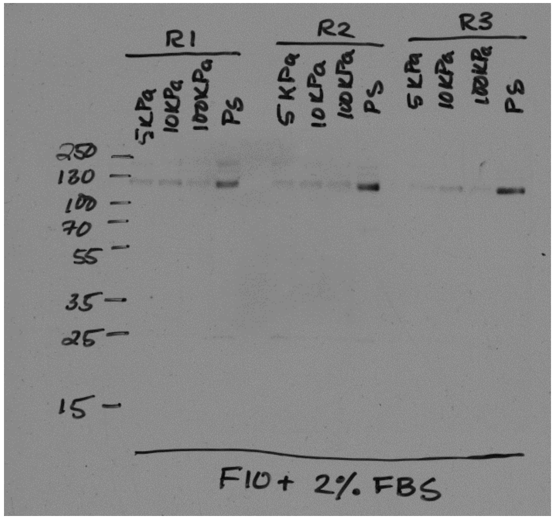

Total Protein (Ponceau)

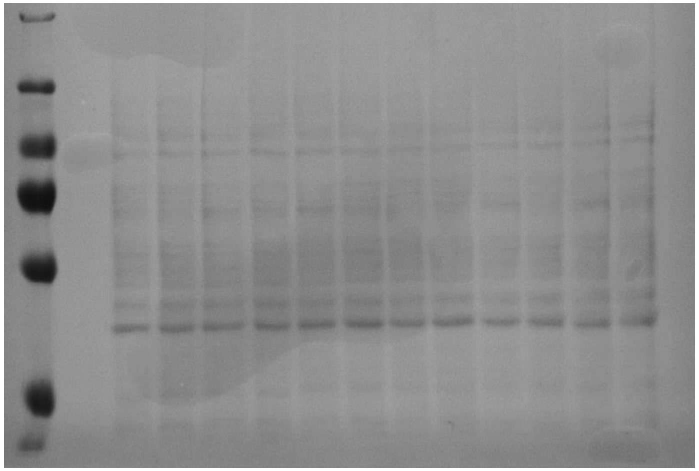

F10 + 10% FBS

EDA-Fn

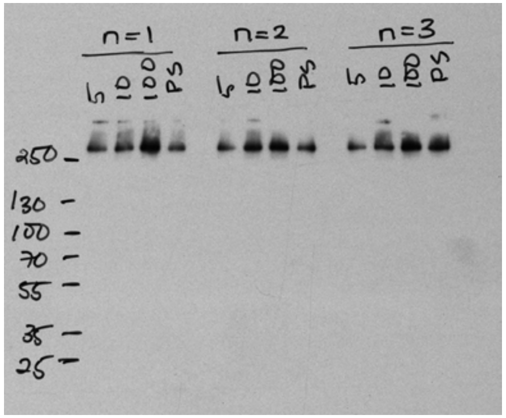

SMemb

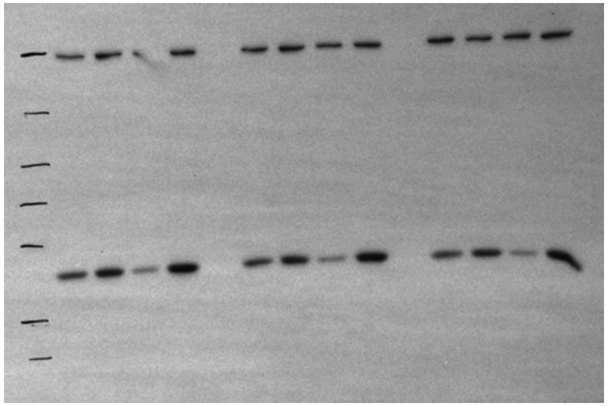

αSMA

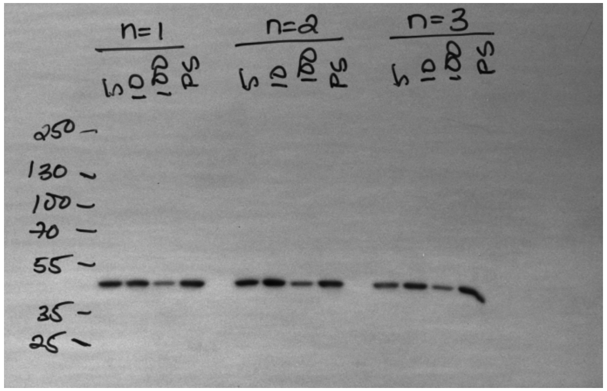

Vimentin

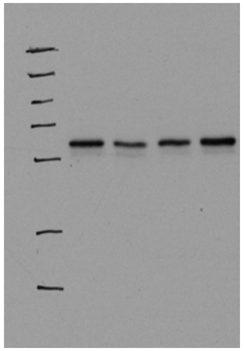

Total Protein

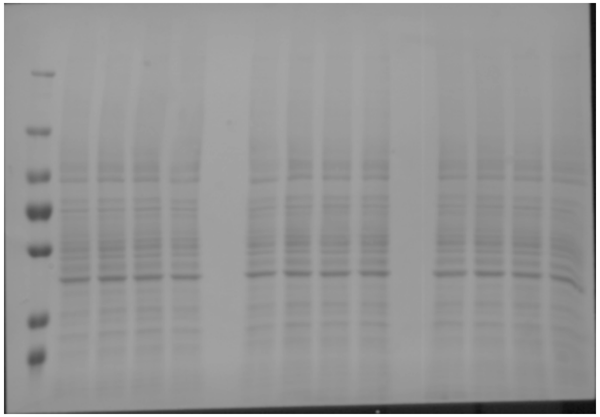

DME/F12 + 10% FBS

EDA-Fn

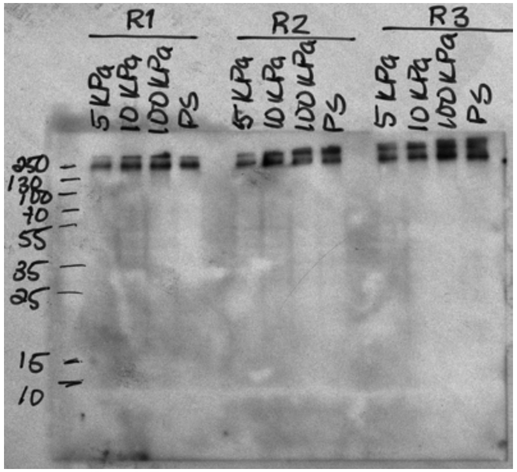

SMemb

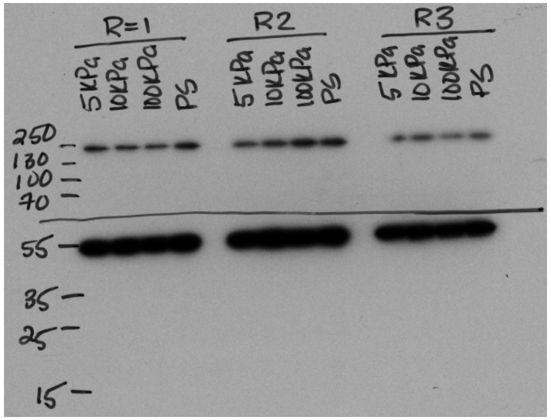

αSMA

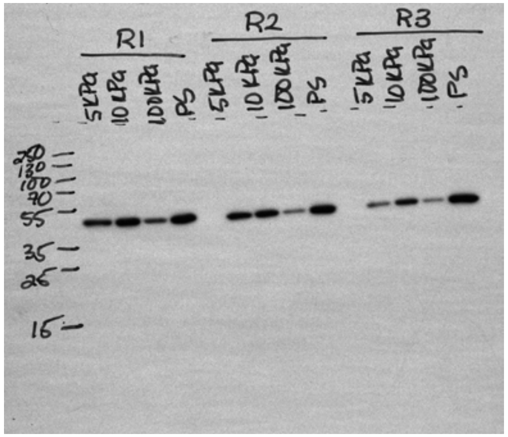

Vimentin

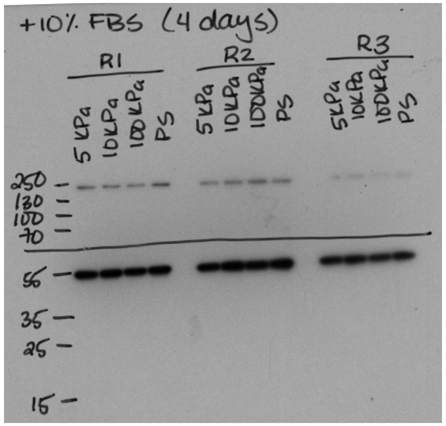

Total Protein

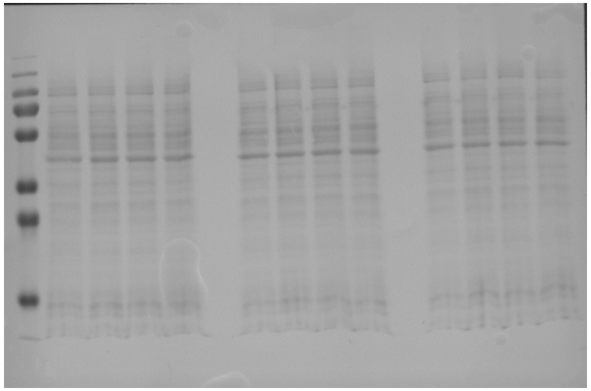

Supplement: Supplementary file 1 — Supplementary Files [file 41598_2019_49285_MOESM1_ESM.pdf]
